# The Physician

**Published:** 1916-11

**Authors:** A. B. Denton

**Affiliations:** 220 Formwalt St., Atlanta, Ga.


### THE PHYSICIAN.

Every labor derives its justice and dignity from its result, and unless there is a result, the labor has been in vain, so it is interesting to note through history, the influence of medical science on the moral and spiritual development through the ages. While in former times, medicine was looked upon as an art, to-day we find medicine not only an art but a science as well. While in former times, we find that the name "physician" suggested to the public mind, craft and guile, to-day the name suggests those to whom we instinctively turn, whose counsel is sought, eager to give time and services to mankind. As the highest aim of medicine is the perfection of mankind, so the highest aim of the physician is to heal, thus we find that the true physician does not look upon his profession as a trade or a business, but more as a sacred vocation and calling. In him, we find one who is neither a fanatic or an egotist, but an individual of calm and sound judgment, treating every subject that arises fairly and logically, leaving no room for caviling. We find nothing in his life or utterances that is self-centered. If he boasts, his boasting is not of personal vanity, but rather a simple and undisputable statement of facts that need to be made. Shakespeare has said of the physician, "Who chooses medicine, must give and hazard all he has." Of medicine, Descartes has said, "If it be at all possible to ennoble mankind, it will only be through medicine." It is a profession that calls for the brains and heart of the greatest men. Men of great strength of character and ripe intelligence, those in whom we find that perfect harmony of heart and brain.

A. B. DENTON.

220 Formwalt St., Atlanta, Ga.

